# Feasibility of remote measurement in intensive longitudinal data collection for rheumatoid arthritis patients commencing a new treatment

**DOI:** 10.1093/rap/rkaf078

**Published:** 2025-07-07

**Authors:** Hsiu Yen Tung, James Galloway, Faith Matcham, Amy Boalch, Ginny Shand, Sam Norton

**Affiliations:** Department of Psychology, King’s College London, London, UK; Centre for Rheumatic Disease, King’s College London, London, UK; School of Psychology, University of Sussex, Brighton, UK; Centre for Rheumatic Disease, King’s College London, London, UK; Department of Psychology, King’s College London, London, UK; Department of Psychology, King’s College London, London, UK; Centre for Rheumatic Disease, King’s College London, London, UK

**Keywords:** rheumatoid arthritis, EMA, Fitbit, longitudinal, intensive monitoring, wearables, ambulatory assessment, somatic symptoms, feasibility, acceptability, qualitative

## Abstract

**Objectives:**

This article aims to evaluate the feasibility and acceptability of intensive assessment of symptoms in RA patients starting a new biologic treatment.

**Methods:**

Participant symptoms and experiences were collected six times a day for 14 days and once a day for 16 days in a single cohort. Wearable devices were also given to participants to track sleep and physical activity. Qualitative interviews were conducted to provide feedback regarding the acceptability of methods. Recruitment and completion rates were used to test for feasibility. The mean and variability of data for each day were calculated to reflect on data quality. Qualitative interview data were analysed by deductive thematic analysis.

**Results:**

Of the 110 patients approached, 27 (15.5%) could not be contacted and 12 (14.5%) were excluded due to meeting exclusion criteria. Of 71 contactable and eligible participants, 31 (43.7%) joined the study. The survey completion rate was 74.6% (1943/2604) for the first 14 days, ranging from 10.7 to 100%. Completion rates for days 15–30 ranged from 60 to 83%. Mean levels of severity of symptoms (pain, fatigue, joint stiffness) showed a decrease after treatment, as expected. Qualitative interviews demonstrated that participants reported a positive experience that was not overly burdensome. Surveys were described as quick and easy to complete, but repetitive for some participants.

**Discussion:**

Recruitment and completion rates were acceptable and comparable to similar studies in the field. Qualitative analysis showed largely positive reviews from participants with feedback mainly focusing on survey timings.

Key messages

- The recruitment rate for a month-long intensive monitoring study consisting of 100 surveys and a wearable device was acceptable (28.1%).
- The completion rate was similar to other shorter ecological momentary assessment studies demonstrating the acceptability of study methodology to patients.
- Qualitative interviews demonstrated that intensive monitoring is acceptable to patients.

## Introduction

RA is a chronic autoimmune inflammatory disorder affecting joints in the hands and feet through synovial membrane inflammation [1, 2], with patients experiencing painful swollen joints and irreversible joint damage [3]. The prevalence of major depressive disorder in RA patients is 16.8% [4], three times greater than in the general population [5].

RA symptoms along with other somatic and psychological symptoms fluctuate over time, even throughout the day [6]. Current measurement methodology consists of assessments only during symptom flares or a scheduled appointment with the medical team, meaning fluctuations are undetected. These day-to-day fluctuations in physical and psychological symptoms, as well as sleep, play an important role in the quality of life of patients [7]. To understand these fluctuations, it is necessary to study RA longitudinally with frequent follow-up over periods of days or weeks [8], providing a continuous longitudinal picture of symptom severity. A scoping review of longitudinal studies in musculoskeletal disorders with follow-ups at least every 14 days [9] found that most studies include no more than three symptoms, limiting the scope of analysis. Ecological momentary assessment (EMA), particularly using wearable devices, combined with modern multivariate analytical methods offers huge potential in understanding the complex dynamic interplay between symptoms over time. EMA involves intensive, real-time collection of data on individuals’ experiences and behaviours in their natural environments. When combined with wearables, EMA enables continuous and objective monitoring of symptoms, physiological variables and contextual factors capturing within-person variability over time. This allows better understanding of the complex and dynamic interplay between symptoms, identifying temporal patterns, causal relationships and potential intervention targets with high ecological validity.

EMA involves repeated sampling throughout the day in the participants’ natural environment to ensure ecological validity and to avoid recall bias [10]. EMA is uniquely suited to collect data from RA patients to study both within-day and between-day symptoms. Complementing EMA with wearable devices capturing physical activity presents the opportunity to collect objective data with minimal additional burden for participants. Studies have demonstrated good feasibility and acceptability of wearables in inflammatory arthritis populations [11, 12] and suggest step counts could accurately detect flares in disease activity. Furthermore, it is feasible and advantageous to monitor RA patients outside of clinical appointments, which EMA surveys and wearables can offer [13]. Chronic arthritis patients have also reported that the use of mobile applications to help support active disease management such as reporting symptom data to clinicians are favoured [14]. However, currently, no studies have considered the feasibility of a combined approach of symptom EMAs and wearables in rheumatologic outpatient samples. A qualitative study carried out on RA patients and rheumatology healthcare professionals [15] found that remote measurement for patient-reported outcomes and ambulatory measurement is easy and allows for more flexibility and control for patients, lending credence to this methodology. Participants have also described current follow-up care as burdensome.

This study evaluates the feasibility and acceptability of intensive monitoring during new biologic treatment using a combined approach involving SMS-based symptom EMA and wearable-based physical activity monitoring. The research questions were:

- Are people with RA willing to participate in a study including symptom EMAs and ambulatory assessments with a wearable device during the initiation of new biologic treatment?
- Is adherence to an intensive monitoring protocol sufficient to allow data analysis?
- Does the intensive monitoring protocol allow for detection of changes in somatic and psychological symptoms?
- What are the views and experiences of study participants regarding intensive monitoring during the initiation of new biologic treatment?

## Methods

### Study design

This was a 30-day single-arm longitudinal observational study. Data were collected using surveys and a wearable device (Fitbit Charge 4; Fitbit, San Francisco, CA, USA). Participants were given the opportunity to participate in a qualitative semistructured interview to provide feedback on the study. Prior to recruitment of the main sample, a pilot test of the entire recruitment and study process were carried out on three participants.

To determine willingness to participate, recruitment rates and EMA survey completion rates for each participant were assessed. This is in line with guidance on the conduct of feasibility and pilot studies [16]. It is recommended that survey response rates in EMA studies should be at least 80% [17] and are typically ≈85% in chronic pain populations [18].

Ethics approval was granted by Health Research Authority and Health and Care Research Wales approval by the Riverside Research Ethics Committee (IRAS ID: 245789).

### Sample

Participants were recruited from rheumatology clinics at King’s College Hospital (KCH), Denmark Hill, London, from February to September 2021. Inclusion criteria were age ≥18 years, clinical diagnosis of RA, commencing a new biologic treatment, willing and able to give informed consent to participate and fluency in the English language. Exclusion criteria were not living in the UK during the study period, no access to a smartphone and no internet access. Patients starting on biologics were chosen because of the current active disease, which provides usable symptom severity data.

To assess the feasibility of intensive monitoring, a target sample size was set at 30 based on the precision with which key parameters of interest could be estimated [19]. This is in line with previous pilot and feasibility studies [19] and allows for an estimate of the recruitment rate with a 95% CI with maximum width of ±15%, assuming at least a 30% willingness to participate.

### Materials

#### Baseline questionnaire

Participants completed a questionnaire including basic demographic information (e.g. age, gender, and disease duration) and patient-reported outcomes, including the Musculoskeletal Health Questionnaire (MSK-HQ) [20], Patient Health Questionnaire 2 (PHQ2) depression screen (97% sensitivity, 67% specificity) [21] and Generalized Anxiety Disorder 2 (GAD2) anxiety screen (86% sensitivity, 83% specificity) [22]. The MSK-HQ was shown to have a high test–retest reliability (0.73) and validity via correlation with other similar tests such as the HAQ [23] and is not disease specific. The PHQ2 and GAD2 also offer good test–retest reliability (0.86 and 0.69, respectively), and both construct and criterion validity [24].

#### Daily questionnaires

The survey consisted of two parts, a symptom EMA sent five times daily at 09:00, 11:00, 13:00, 15:00 and 17:00 and a longer survey sent at 20:00. The survey at 20:00 included the symptom EMA plus questions regarding physical activity, sleep, quality of life and Fitbit usage. The EMA assessed 11 somatic and psychological symptoms scored on a 0–10 numerical rating scale (NRS). These included three somatic symptoms (joint pain, joint stiffness, fatigue), four positive affects and four negative affects. These questions are in Supplementary Data S1 and S2, available at *Rheumatology Advances in Practice* online. The EMA survey was completed six times per day for the first 14 days. For the last 16 days, only the longer 20:00 survey was sent. This decrease in quantity of surveys per day was due to a worry about patient burden.

#### Fitbit

Participants were given a Fitbit Charge 4 at the start of the study to objectively measure sleep and physical activity. According to a study comparing various activity monitors [25], patients rated the experience with Fitbit as 4.7 out of 5, suggesting good satisfaction. A previous systematic review of 67 studies assessing the accuracy of Fitbit on step counts [26] demonstrated consistent evidence that Fitbit provides acceptable accuracy. Another review [27] compared the accuracy of Fitbit with polysomnography and found that Fitbit distinguishes between sleeping and waking times at an accuracy level of 0.81–0.91. As such, the Fitbit Charge 4 was deemed appropriate for collecting objective information regarding physical activity and sleep.

### Procedures

Participants were recruited from rheumatology clinics at KCH. Eligible patients who were due to start a new biologic treatment were identified during multidisciplinary team meetings and subsequently approached via telephone.

Potential participants were provided with an information sheet by e-mail and a link to an electronic consent form. Consenting participants were asked to complete a baseline questionnaire. They were provided with a Fitbit that they could keep after the end of the study. Processed data from the Fitbit was remotely downloaded after the 30-day period using the provider API key. Data used in the analysis were the daily totals, but data for other intervals was also stored, including estimated steps, calories burned and sleep in intervals of 1 min and heat rate every 5 sec.

Participants provided researchers with their start date for the new biologic treatment to determine when surveys were to be sent. Surveys started approximately 3 days before commencement of the new biologic therapy to collect data pre-exposure and were sent via Survey Signal text message with a link to an online questionnaire. This was followed by 27 days of post-exposure data. Some estimated start dates provided by patients were inaccurate, thus a complete 3-day pre-exposure dataset could not be collected. Participants were expected to wear the Fitbit throughout the 30 days to objectively measure sleep and physical activity. Participants received six surveys daily for the first 14 days, then one survey daily for the remaining 16 days, totalling 100 surveys. This was designed to minimize patient burden while still providing a detailed picture of daily fluctuation. No reminders were given to the participants to complete the surveys.

All participants were contacted towards the end of the study to invite them to participate in an interview to discuss their study experience. Those who consented were interviewed via Microsoft Teams or Zoom. Meetings were recorded and transcribed. A COREQ Checklist (see Supplementary Material, available at *Rheumatology Advances in Practice* online) has also been included to enhance the quality and transparency of the qualitative component.

### Analysis

To establish the feasibility of the protocol, recruitment and survey completion rates of each participant were assessed. The number of participants approached and recruited and the reasons for non-recruitment of potential participants were investigated. The completion rate of EMA surveys by time of day was assessed over the first 14 days and retention in the study assessed over the entire 30-day period of data collection. The overall completion rate was calculated for the first 14 days by dividing the total number of responses to the EMA surveys by the total number of possible responses (i.e. 6/day × 14 days = 84 total surveys per participant). EMA surveys can only be submitted upon completion of every question. Wear time for the Fitbit was calculated for the number of days over the entire 30-day study period that had at least 15 active minutes recorded on the Fitbit.

Deductive thematic analysis was performed on the qualitative data by two researchers (A.B. and G.S.). The analysis followed procedures outlined by Braun and Clarke [28], using predefined codes and themes adapted from a pre-existing framework, the theoretical framework of acceptability (TFA) [29]. This framework was adapted to focus on the acceptability and feasibility of the methods used in the present study.

Researchers familiarized themselves with interview transcripts before creating a reference code book (see Supplementary Data S3, available at *Rheumatology Advances in Practice* online). Two researchers (A.B. and G.S.) independently coded one transcript to assess the fit of the code book with the data. Subsequent changes to the code book were agreed upon by both researchers. The researchers then independently coded a selection of interview transcripts with post-coding reconciliation conducted by a third researcher. Codes were organised into ‘themes’ and ‘subthemes’.

## Results

### Recruitment rate

The average time from healthcare professionals identifying potential participants to recruitment was ≈5 days. Participants commenced daily data collection between 1 day and ≈2 months later (mean 3 weeks), depending on when biologic treatment started.

**Figure 1.**
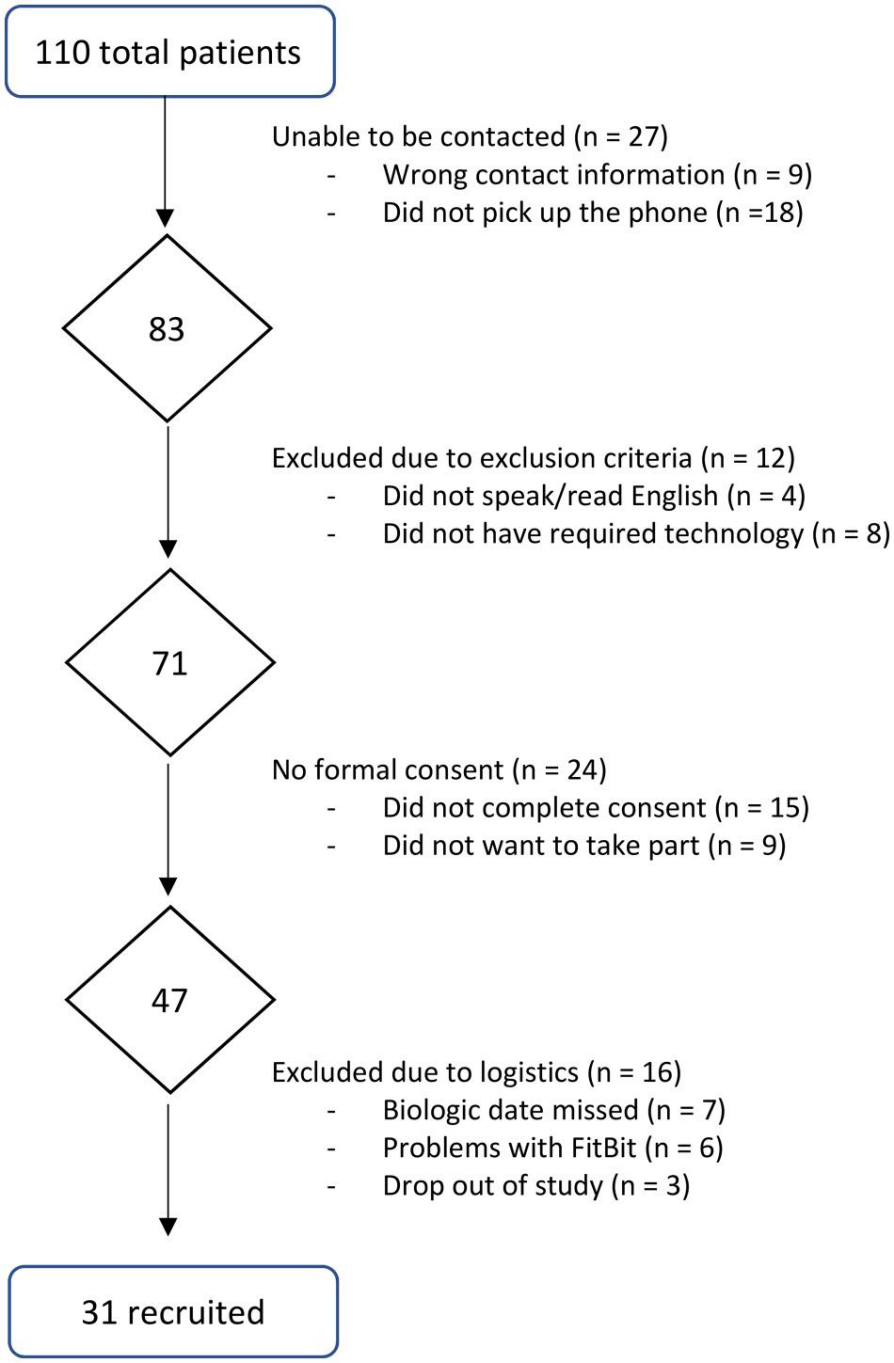
Flow chart of participant recruitment

In total, 110 potentially eligible participants were identified and contacted. Of these, 31 [28.1% (95% CI 22.4, 40.4)] were recruited and commenced daily data collection. Figure 1 shows a flow chart detailing the reasons for non-participation in the study. The baseline demographic information for the 31 participants who commenced daily data collection is given in Table 1, separated into the total sample and those that participated at the interview.

**Table 1.** Sample characteristics.

Characteristics	Interview subsample (*n* = 15)	Total sample (*N* = 31)
Age, years, mean (s.d.)	52.53 (12.9)	50.16 (14.7)
Gender, *n* (%)		
Female	13 (86.67)	26 (83.87)
Male	2 (13.33)	5 (16.13)
Ethnicity, *n* (%)		
White British	9 (60.0)	19 (61.3)
Asian British	3 (20.0)	7 (22.6)
Black or Black British	2 (13.3)	4 (12.9)
None provided	1 (6.7)	1 (3.2)
Previous experience using wearable tracker, *n* (%)	13 (86.7)	22 (71.0)
Disease duration, mean (s.d.), days	159 (53)	184 (81)
28-joint DAS, mean (s.d.)	5.6 (0.5)	5.2 (0.9)
MSK-HQ, mean (s.d.)	27.9 (11.6)	34.2 (14.2)
PHQ2, mean (s.d.)	3.2 (1.1)	3.8 (1.3)
GAD2, mean (s.d.)	2.7 (0.8)	3.0 (1.1)

Of the 110 patients approached, 27 [15.5% (95% CI 9.3, 23.6)] were not contactable, including 9 patients with incorrect contact details and 18 patients who did not answer the phone despite multiple attempts (three calls at various times over 3 weeks). A total of 83 potential participants were contacted via telephone. Of these, 12 [14.5% (95% CI 7.7, 23.9)] were unable to proceed due to exclusion criteria. Of the 71 eligible participants, 24 [33.8% (95% CI 23.0, 46.0)] did not consent to participate, either explicitly declining or indicating interest but not completing the consenting process despite at least one reminder. In addition, while a total of 47 consented to participate, 13 of these [27.7% (95% CI 15.6, 42.6)] participants withdrew consent prior to starting data collection. Of these, six had issues using the Fitbit (two reported skin reactions; one reported lymphoedema, preventing wearing of the Fitbit; and three could not operate the Fitbit). The remaining seven were unable to commence daily data collection due to initiation of biologic treatment before it was possible to begin data collection. A further three failed to commence daily data collection for undisclosed reasons.

### Survey completion rate

**Figure 2.**
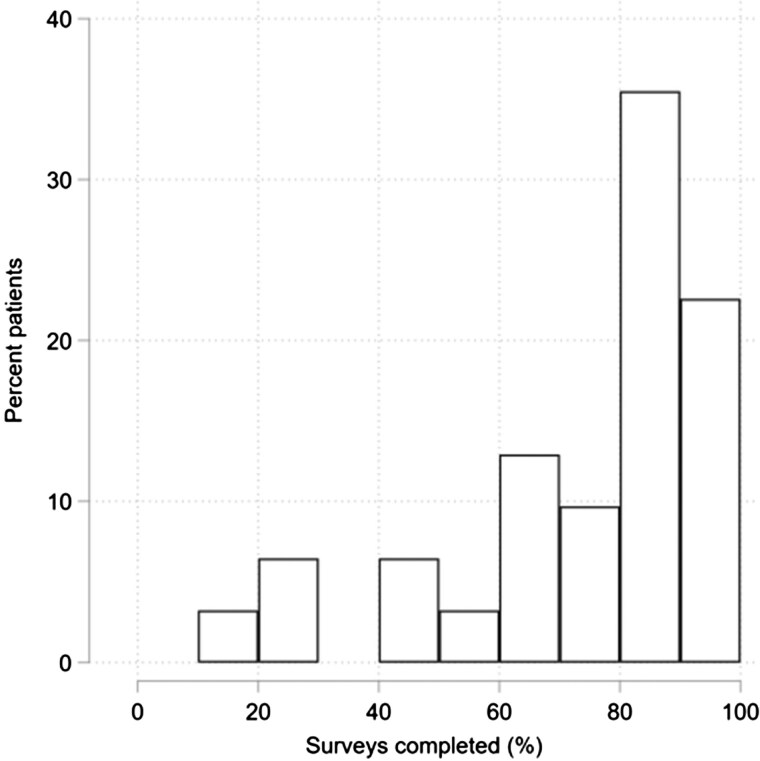
Percentage of EMA surveys completed per patient (*N* = 31)

**Figure 3.**
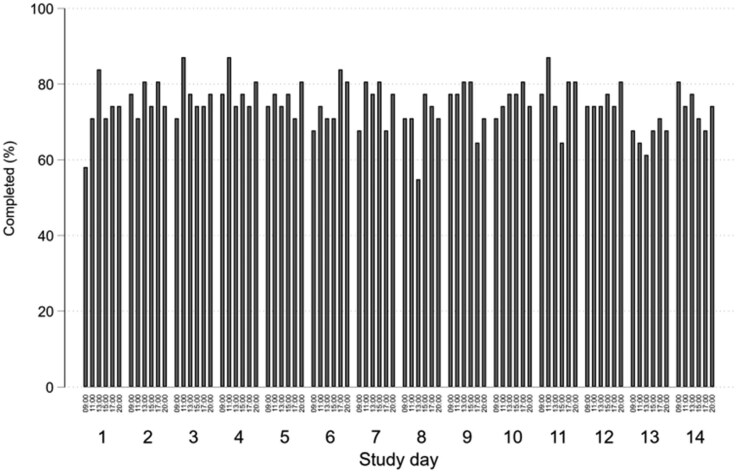
Percentage of EMA surveys completed by day and time of day

**Figure 4.**
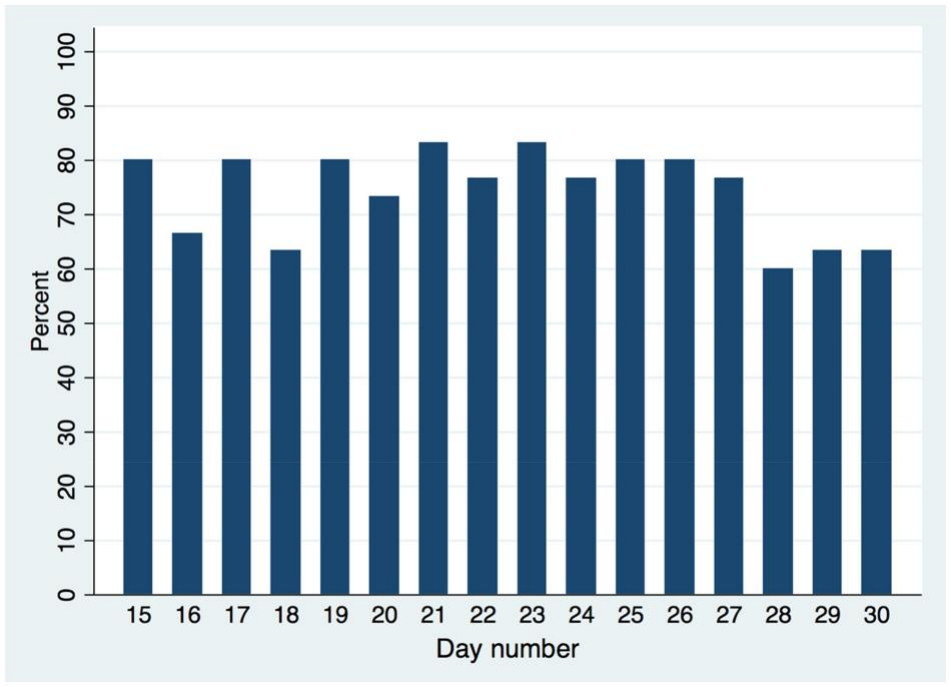
Completion rates for days 15–30 of the study

For the 31 participants who commenced daily data collection, the completion rate across all surveys was 1943/2604 [74.6% (95% CI 72.9, 76.3)] for the 14 days of EMA surveys. Completion rates varied across individuals, from a minimum of 10.7% to a maximum of 100% [Fig. 2; median 84.5% (interquartile range 66.7–89.3]), with ≈60% completing at least 80% of the surveys. A total of 26/31 [83.9% (95% CI 66.3, 94.5)] participants completed surveys on all 14 days of the EMA surveys, with no patients completing surveys on <7 days (Fig. 3). Completion rates by time of day over the first 14 days was relatively stable, with most being >60% but rarely exceeding 80%. The completion rates for each time of day were consistent, ranging from 72.4% for the 09:00 survey to 76.5% for the 11:00 survey. Over the last 16 days of the study, when only one survey was sent each day, completion rates ranged from 60% to 83%, with the last 3 days of the study showing a distinct reduction (Fig. 4).

**Figure 5.**
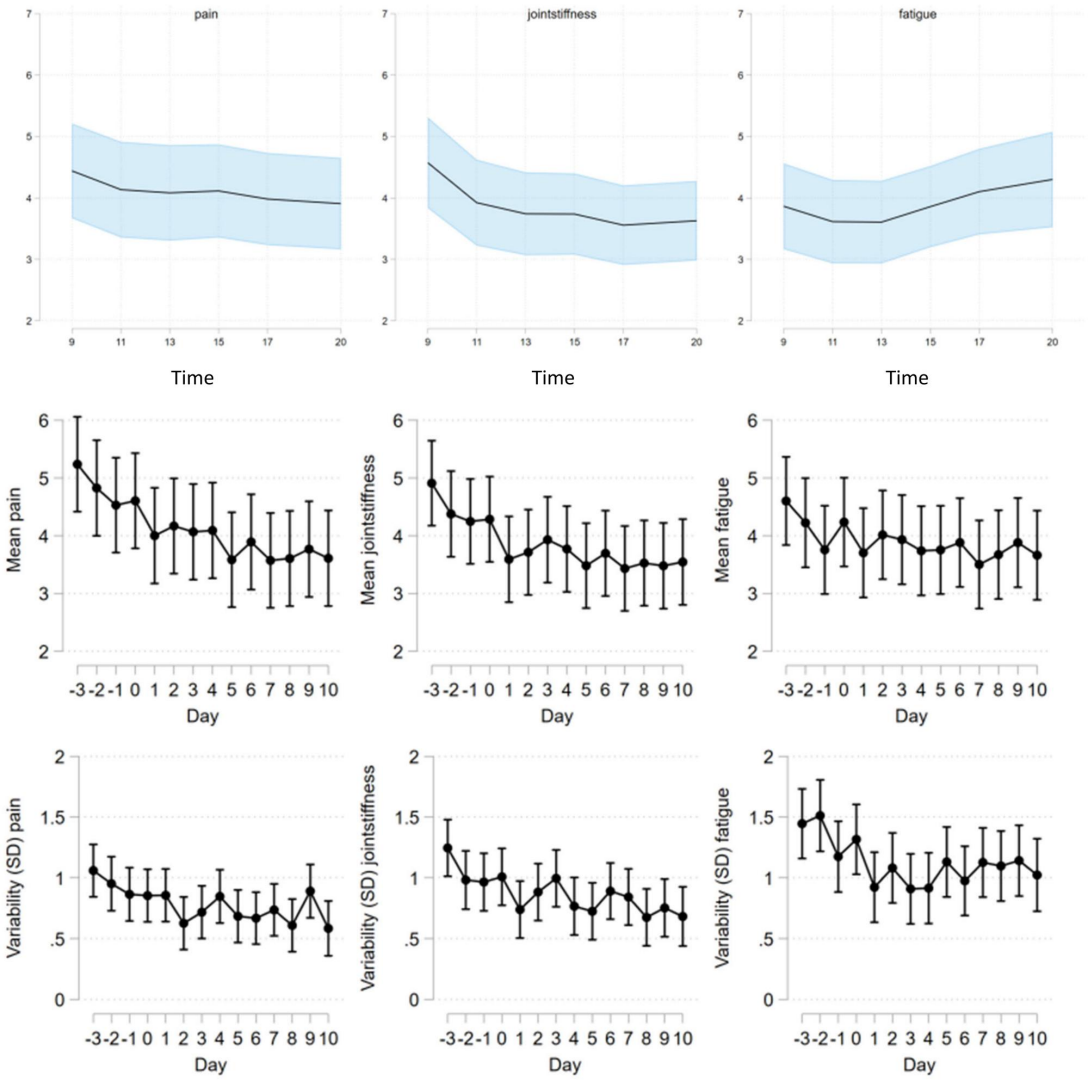
Mean and variability levels of somatic symptoms

Looking at the quality of the data, Fig. 5 shows a fluctuation of the mean levels of the three somatic symptoms and positive and negative affect. Days −3 to −1 were pretreatment days and day 0 was the first day of a new biologic treatment. There was a significant *P*-value in the mean and variability differences for all somatic symptoms between pre- and post-treatment days, while most were insignificant for affect, which can be seen in Supplementary Tables S1 and S2, available at *Rheumatology Advances in Practice* online.

### Wearable device

For the 31 participants, 30 [96.8% (95% CI 83.3, 99.9)] were able to provide data using the wearable device on at least 1 day. The total number of days when the wearable device was worn was 811 (87.2%) of a total possible number of 930 days (i.e. 30 days × 31 participants), with an average active wear time per day of 242 min. Fitbit recording of overall time active and time fairly/very active agree well with the self-reported highest level of physical activity for that day (Supplementary Fig. S1, available at *Rheumatology Advances in Practice* online). Nighttime sleep was recorded on 616 days (66.2%). The mean level of sleep recorded on the Fitbit was 407 min (s.d. 83) compared with 398 min (s.d. 81) by self-report, with an intra\-class correlation of 0.43 (95% CI 0.32, 0.53) (Supplementary Fig. S2, available at *Rheumatology Advances in Practice* online).

### Qualitative results

A total of 15 participants from the main study consented to and completed qualitative interviews (Table 1). Deductive coding used three pre-specified themes (Supplementary Data S3, available at *Rheumatology Advances in Practice* online) adapted from the TFA [29]: ‘affective attitude’, ‘perceived burden and costs’ and ‘perceived effectiveness’. During coding, subthemes regarding data capture and real-world use were created. Quotes from participants are presented with the participant ID to support and illustrate themes.

#### Affective attitude

Overall, participants expressed positive feedback regarding the study, with multiple participants sharing that the study experience benefited them personally (*n* = 10/15).

‘I didn’t expect to get personal gains from it but I have’. (Participant 13)

Some participants reported the study provided them with designated time to reflect on their symptoms (*n* = 3/15).

‘I was able to spend that time reflecting…I’ve actually really enjoyed it’. (Participant 3)

One participant remarked that ‘the Fitbit prompted me about my health more than the survey prompted me about my health’ (Participant 13). This was echoed by many other participants who reportedly learned a lot about their sleep quality and physical activity levels from the Fitbit (*n* = 8/15).

#### Perceived burden and costs

Most participants reported the survey completion was not overly burdensome and that they were quick and easy to complete (*n* = 10/15).

‘It just used to come in on your phone and then you click the link, and it was yeah…you just fill it in’. (Participant 8)

These comments were in line with the high completion rate of surveys from all participants. Four participants mentioned that the surveys presented some burden, as the questions became boring; however, this did not deter patients from completing the survey. Some participants (*n* = 4/15) suggested that a reduced frequency of surveys, such as four times daily, would be more appropriate, especially for those working in full-time employment.

‘It was irritating, but I did it’. (Participant 6)

‘The six times a day at first…it was quite repetitive. If it was every four hours I might be putting something different….because I’m working and doing the same thing for 8 hours continuously…every two hours was a bit unnecessary for me’. (Participant 14)

For most, the study seemed to become less burdensome the more familiar participants became with the survey. The consensus was that frequent monitoring was manageable and became increasingly straightforward with time (*n* = 13/15).

‘Once you’ve done a couple they’re so quick and easy’. (Participant 2)

The Fitbit was considered low burden. Participants reported it was ‘straightforward’ (Participant 13) and comfortable to wear (*n* = 5/15). Some participants reported that they would continue to wear their Fitbit after the study (*n* = 7/15). Tracking one’s health was not accompanied by an increase in health concern or worry, with many patients reporting they were already very aware of their medical condition (*n* = 11/15).

‘I know I’ve got this condition, I’ve had it for years, it’s got worse…I have to deal with it’. (Participant 15)

#### Perceived effectiveness

##### Data capture

The timing of the surveys was considered particularly important, and participants made various suggestions to change survey timings. One participant reported the first survey (09:00) was too late in the day to capture morning stiffness. Conversely, another reported missing survey completion due to sleeping in beyond the first survey.

‘It always said in the last hour, which didn’t capture morning stiffness, because if I had got up at 6am and then you sent the first one at 9am, the last hour is long after that stage’. (Participant 2)

‘There was a few that I missed in the morning…on my days off where I didn’t get up until about 11am’. (Participant 1)

A common suggestion to improve the survey data collection process was the addition of a free-text box to provide context to the survey responses, for example, to explain why mood was low at a certain time if this was not due to RA symptoms (*n* = 3/15).

Most people reported wearing the tracker all day, but a common concern among participants was getting the device wet, despite knowing the device was waterproof. This meant for some, the device was removed when washing up or bathing (*n* = 7/15).

##### Real-world use

Remote monitoring of health was mostly reported as reassuring for patients (*n* = 4/15). It was reported that this could benefit them not only through activating their own personal behaviour change (*n* = 11/15) but also to provide their healthcare team with better insights into the patients’ health (*n* = 6/15).

‘I think it’s important to do this and you have the record because when we come to clinic and we’re so anxious about feeling well, even if you’ve been feeling rotten the rest of the weekend you come in and say—yeah, yeah, I’m OK’. (Participant 11)

‘It can just give a bit more understanding of what you’re doing, things you can improve and so forth’. (Participant 4)

Patients expressed that frequent monitoring through a wearable device and surveys could be a holistic approach to healthcare services, giving a better picture of the patients’ day-to-day well-being (*n* = 5/15).

‘I think it would help the medical team see the real world of how people are living’. (Participant 13)

## Discussion

This study demonstrates the feasibility and acceptability of an intensive monitoring protocol in people with RA around the time of initiating biologic therapy, but it also reveals significant challenges in recruitment and retention. Of the 110 patients approached, only 28.1% were successfully recruited, with approximately one-third of those who initially consented [13/47 (27.7%)] withdrawing before starting the study. This suggests that despite initial interest, barriers remain that may deter ongoing participation, highlighting the need for strategies to better engage and support participants during recruitment to studies involving intensive symptom monitoring. Additionally, while 74.6% of the 2604 EMA surveys were completed, this completion rate reflects a considerable decrease from the invited population. Taken within the context of the relatively high burden on the respondent with six surveys per day, this the completion rate is positive but nevertheless demonstrates room for improvement in supporting adherence to intensive monitoring protocols.

Qualitative interviews conducted with participants showed that 10 of 15 found the study acceptable. No major concerns were raised, although feedback about the repetitiveness and timing of surveys suggests that adjustments to these elements could improve participant experience and adherence. Importantly, the perspectives of those who withdrew or did not complete interviews remain underrepresented. The relatively low number of participants who completed interviews (15/31) further highlights the challenge of maintaining engagement in research. Of the 31, only 18 participants were contacted and the rate of participation (15/18) was very high. Participation was partly influenced by decisions not to interview those who withdrew and the availability of researchers to conduct interviews. Future studies should aim to engage those who struggle to participate more effectively and ensure nested qualitative studies are sufficiently resourced. By addressing these barriers and refining the protocol, future research can better ensure that intensive monitoring during biologic treatment is both feasible and acceptable to a broader range of participants. In a similar study where interviews were carried out with nurses, patients and rheumatologists about the perceptions on mobile health applications [30], the necessary components to making an application like this successful is to avoid long-term poor compliance, finding the correct target audience, providing a framework for users and making sure the application is easy to use.

Despite the above considerations, it is important to note that a recruitment rate of approximately one in three is in line with other feasibility studies, highlighting the challenges of recruitment to clinical studies. Furthermore, recruitment issues are common in health research, with half of studies requiring funding extensions for this reason [31]. The recruitment strategy here was also a convenience sample rather than recruitment until data saturation and thus may represent a potential selection bias. Any future study will need to account for these issues when planning recruitment, which may mean that the sample is not representative of the RA population.

It is important to note that ≈1 in 10 people who were contactable did not have a smartphone and a further 1 in 10 who were provided with a Fitbit were unable to use it. Technology usage poses an issue possibly because of the generally older population that RA samples are derived from. Smartphones were predicted to have ≈6.3 billion users in 2021, and the older population of ≥51 years use smartphones ≈100 min/day [32]. Therefore, we would anticipate that these issues will diminish with time. The study design and burden are generally acceptable for potential participants, and in ideal clinical situations with face-to-face recruitment, the recruitment rate may be expected to be higher. However, it is important to note that only 3 of 47 participants were excluded from this study due to a lack of technological literacy resulting in them being unable to use the Fitbit.

The study also indicated that an intensive monitoring protocol was acceptable to most patients that were recruited. In total, 30 of 31 patients provided at least 3 days of data and 30 of 31 were retained at day 30, with around three-quarters of all surveys sent being completed. However, there is a need to consider how response rates can be improved, given rates of >80% have been deemed as acceptable for EMA studies to provide robust data for analysis [17, 33]. Our response rate was also lower than reported in a meta-analysis of chronic pain patients [18], where the average completion rate was 85%. This is potentially due to combining EMA surveys with a wearable device and with our rate being comparable with that reported in a review by Wen et al. [34], which found a completion rate of 73% when EMA is combined with a wearable device.

Qualitative data demonstrated that patients valued being able to monitor their own health in real-world settings. This is echoed by a qualitative study on patients using different self-monitoring ‘health apps’ [35], which reported a benefit for patients in tracking symptoms in real-time. While intense data collection came with some burden to patients, there was consensus that the insights it could provide their medical team would be useful. Overall, Fitbit was deemed to be straightforward and easy to use by patients; however, these findings may be subject to selection bias due to patients with technological barriers not enrolling in the study. In a systematic review investigating barriers in remote measurement [36], one of the top themes is convenience and accessibility. The use of easily accessible surveys on a smartphone and passive data measurement with a Fitbit fits into this. In further analysis of the data collected in this study we will more robustly evaluate daily and within-day variability in symptoms to ascertain whether intensive monitoring with only a single assessment per day is valid and whether a specific time of day appears more appropriate.

While this remote monitoring approach may be used to evaluate treatment response, it is important to note that this was not the intention of this article, which focused on the feasibility of data collection. Data on response must be interpreted with caution, as clinical responses from biologic treatment vary across drug classes and may range from a few weeks to months [37]. Therefore, no significant improvements in symptom severity may be detected during this study period. There is also a possibility of confounding factors, including placebo response, that affect the symptom severity at the beginning of the study period. It is also important to mention that this is the first study that has explored such intensive monitoring measures alongside a wearable device, and only 30 participants were recruited. This means that further research needs to be carried out regarding the feasibility and usability of these methodologies to affirm the result. Furthermore, the recruitment rate and compliance rate in this study also need improvement, which suggests that a change in study design may be beneficial. It is also important to note that the different biologic treatments that each individual receives will have a different response time as well, which is an important feature to investigate in future research.

In conclusion, intensive monitoring with a combined EMA and wearable was shown to be feasible and acceptable to RA patients at the time of starting biologic treatment. The recruitment rate was comparable to other studies of similar design. Data quality was comparable to studies in other populations and sufficient for analysis of trends over time. The use of such protocols in research and clinical practice potentially allows for far greater insights into symptom fluctuations and trends over time both at the group and individual level. However, similar studies need to be repeated to allow for generalisations to be made and to fine tune the methodology to allow for a higher rate of participant compliance.

## Supplementary Material

rkaf078_Supplementary_Data

## Data Availability

The data underlying this article cannot be shared publicly due to the privacy of individuals who participated in the study and also the fact that they are National Health Service patients who were recruited to the study. The anonymised version of the data will be shared upon reasonable request to the corresponding author.
