# Supplementary material for: Feasibility of remote measurement in intensive longitudinal data collection for rheumatoid arthritis patients commencing a new treatment
Source: Rheumatol Adv Pract. 2025 Jul 7;9(3):rkaf078. doi: 10.1093/rap/rkaf078 (PMC12375405; doi:10.1093/rap/rkaf078)
Supplement: rkaf078_Supplementary_Data [file rkaf078_supplementary_data.zip › COREQ Checklist_EMA study.docx]

| **Domain 1: research team and reflexivity** | | |
| --- | --- | --- |
| **Personal characteristics** | | |
| 1. Interviewer/facilitator | Which author(s) conducted the interview or focus group? | Three researchers carried out the interviews (HT, AB and GS) |
| 2. Credentials | What were the researcher’s credentials? (e.g. PhD, MD) | - HT was a PhD student and these interviews were carried out as part of his thesis. - AB was on her research placement during her Foundation Years as a doctor. - GS completed their MD in health psychology within 3 months of the interviews. |
| 3. Occupation | What was their occupation at the time of the study? | - HT was a PhD student. - AB was a medical doctor. - GS was a master’s student at the time of the research |
| 4. Gender | Was the researcher male or female? | - HT is male - AB and GS are female |
| 5. Experience and training | What experience or training did the researcher have? | - HT finished two masters and was on his 3^rd^ study in his PhD. - AB helped out in various research projects in the Rheumatology office. - GS had undertaken training in qualitative research methodologies as part of their BSc in Psychology |
| **Relationship with participants** | | |
| 6. Relationship established | Was a relationship established prior to study commencement? | No prior relationship was established between the researchers and participants. |
| 7. Participant knowledge of the interviewer | What did the participants know about the researcher? (e.g. personal goals, reasons for doing the research) | Participants knew the purpose of the research and that researchers were part of the rheumatology academic research department of King’s College Hospital. |
| 8. Interviewer characteristics | What characteristics were reported about the interviewer/facilitator? (e.g. bias, assumptions, reasons and interests in the research topic) | All three interviewers had an interest in the research. |
| **Domain 2: study design** | | |
| **Theoretical framework** | | |
| 9. Methodological orientation and theory | What methodological orientation was stated to underpin the study? (e.g. grounded theory, discourse analysis, ethnography, phenomenology, content analysis) | The qualitive portion of the research was underpinned by ‘The Acceptability Framework’. Analysis was based on the deductive thematic analysis method. |
| **Participant selection** | | |
| 10. Sampling | How were participants selected? (e.g. purposive, convenience, consecutive, snowball) | A convenience sample was used for the qualitative research, based on those form the main study who agreed to take part in an interview. |
| 11. Method of approach | How were participants approached? (e.g. face to face, telephone, mail, e-mail) | Participants were approached by telephone or email. |
| 12. Sample size | How many participants were in the study? | The main study contained N=31 participants, n=15 participants took part in qualitative interviews. |
| 13. Non-participation | How many people refused to participate or dropped out? Reasons? | n=15 participants did not agree to take part in qualitive interviews. Numbers of direct refusals were not recorded. |
| **Setting** | | |
| 14. Setting of data collection | Where was the data collected? (e.g. home, clinic, workplace) | Interviews took place over tele-conference (Zoom or Microsoft Teams). |
| 15. Presence of non-participants | Was anyone else present besides the participants and researchers? | Only the researchers were present. |
| 16. Description of sample | What are the important characteristics of the sample? (e.g. demographic data, date) | Participant demographic information for qualitive interviews is presented in Table 1. |
| **Data collection** | | |
| 17. Interview guide | Were questions, prompts, guides provided by the authors? Was it pilot tested? | A semi-structured interview guide was used, including open ended questions and additional prompts to be used where required. These were created and reviewed by the research team before commencing interviews. |
| 18. Repeat interviews | Were repeat interviews carried out? If yes, how many? | No repeat interviews were required. |
| 19. Audio/visual recording | Did the research use audio or visual recording to collect the data? | Interviews were audio-recorded. |
| 20. Field notes | Were field notes made during and/or after the interview or focus group? | No. |
| 21. Duration | What was the duration of the interviews or focus group? | Interviews lasted approximately 30 minutes. |
| 22. Data saturation | Was data saturation discussed? | Data saturation was not discussed. All participants interested in taking part in an interview were interviewed. |
| 23. Transcripts returned | Were transcripts returned to participants for comment and/or correction? | Transcripts were not returned to participants. |
| **Domain 3: analysis and findings** | | |
| **Data analysis** | | |
| 24. Number of data coders | How many data coders coded the data? | Two researchers (AB and GS) coded the data. |
| 25. Description of the coding tree | Did authors provide a description of the coding tree? | A coding framework is provided in Supplementary Data S3. |
| 26. Derivation of themes | Were themes identified in advance or derived from the data? | Themes were identified in advance and were based on The Acceptability Framework. |
| 27. Software | What software, if applicable, was used to manage the data? | Microsoft Excel. |
| 28. Participant checking | Did participants provide feedback on the findings? | No. |
| **Reporting** | | |
| 29. Quotations presented | Were participant quotations presented to illustrate the themes/findings? Was each quotation identified? (e.g. participant number) | Demonstrative quotes have been presented throughout the qualitive results section, with participant codes assigned to all participants and used against quotations. |
| 30. Data and findings consistent | Was there consistency between the data presented and the findings? | We aimed to report the study findings in a clear, consistent manner to accurately represent the qualitive data that was collected.  Major themes are clearly presented.  This section also includes discussion relating to sub-themes. |
| 31. Clarity of major themes | Were major themes clearly presented in the findings? |  |
| 32. Clarity of minor themes | Is there a description of diverse cases or discussion of minor themes? |  |
