# Supplementary material for: Feasibility of remote measurement in intensive longitudinal data collection for rheumatoid arthritis patients commencing a new treatment
Source: Rheumatol Adv Pract. 2025 Jul 7;9(3):rkaf078. doi: 10.1093/rap/rkaf078 (PMC12375405; doi:10.1093/rap/rkaf078)
Supplement: rkaf078_Supplementary_Data [file rkaf078_supplementary_data.zip › Interview Topic Guide .docx]

Interview Schedule/Topic Guide

**Process Evaluation Assessments:**

The primary aim of this interview is to evaluate the experience of the participants from the study, and also the feasibility of the schedule for the Patient Reported Outcomes which may improve future studies that utilises Ecological Momentary Assessment (EMA) or daily assessment designs in patients with Rheumatoid Arthritis (RA). Anonymised quotes may be used in internal reports and research purposes. This interview will only be conducted at the end of the study and will be a maximum of 30 minutes. It will also be audio recorded on encrypted voice records and transcribed by a member of the research team to ensure data security and protection.

**Interviewer:** “Thank you for participating in this study. I’d now like to ask you some questions about your experiences during the 3 months of participating, about how you found using the technology, and to understand what we can do better in the future. This interview is being recorded, but you can let me know if you would like the recorder to be turned off at any point. All information you provide is anonymous – I will be writing a report to share with other members of the team, but no identifiable information about you will be included. We also might use some quotes from you in published papers, but these will be anonymised and not connected to you in any way.

**For participants at the end of the study:**

| **Interview Purpose** | **Questions/Prompts for Interviews** |
| --- | --- |
| **General participation experience**   - Identify opportunities for improvement in the study experience | 1. How did you find the experience of participating? 2. Did you find any aspect of the study particularly burdensome? Can you give me some examples of how/when/why?   *Prompt: Did you ever feel you needed to spend more time or effort than you would like to in a specific area of the study?* |
| **Working the study into their daily lives**   - Identify any points where study participation changed behaviour, or contributed to any trouble. | 1. Has taking part in the study fit into your usual routine?   *Prompts: Did you have to change any part of your routine in order to take part? What did you change, and how did you do it?*   1. What could have made it easier for you to fit the study in your daily lives? |
| **Experience with Apps on smartphones**   - Access familiarity with technology coming into the study, and the learning curve needed. - Identify points that could be modified to aid in designing the app | 1. Were you familiar with smartphones before you started using them as part of this study?   *Prompts: Did you own a smartphone before taking part in this study? If so, had you ever used a health tracking application?*   1. Have you used the smartphone or wearable device for any other purpose than how you were asked to use it in the study, i.e. measure your health?   *Prompts: did you use the watch function? Did you use the smartphone as us usually would to make phone calls, browse the internet etc.? Did you link it to any other device?*   1. Is there anything about the study mobile device that you find burdensome or inconvenient?   *Prompts: What was it, and why? How did it affect your participation or routine?* |
| **Experience with MyArthritis App**   - Evaluating how user-friendly the app is. - Identifying where there could be improvements | 1. Have you ever used any app similar to the MyArthritis App before? If yes, what is it and how do you compare it to this app? 2. How easy did you find the interface of the app is to interact with?   *Prompts: Can you manage to find everything that you want to in the app? How would you rate it from 1 to 10. Have there been any problems with trying to use any aspects of the app?*   1. What do you think should be included/excluded from the app? 2. Will you continue to use the app after the study is finished? Do you find the app helpful? |
| **Experience with study surveys**   - Evaluate the participants’ experience in answering the surveys through text message. - Assess the value of using Qualtrics/Surveysignal | 1. How do you find the study’s applications and mobile surveys to use?   *Prompt: Is the method that the survey is sent through text lacklustre? Or is the survey itself lacking and could be improved.*   1. Do you find the frequency of questionnaires acceptable?   *Prompts: How did this app compare to other health management apps you have used? Was the frequency and length of the app-delivered survey appropriate? Would it be better more/less frequent, shorter or longer? Did you have any other problems with the app?*   1. Is it important to you that you can choose when you complete a survey?   *Prompt: Did you find it helpful to be able to postpone completing the survey?*   1. How do you find the online system for completing the surveys? 2. Have you noticed any change in your data usage or battery life since you starting participating? 3. Are you satisfied with the information about the data collected throughout the study?   *Prompts: Would it make you more or less likely to participate in a future study? Do you have experience of using any other apps or symptom feedback systems? If you would look at feedback, how would you like to receive this? Should it be optional? Provided in the app? Should it be visual, with graphs, or verbal feedback? What kinds of graphs are useful? How often should it be provided?* |
| **Experience with wearable devices.**   - Assess familiarity with wearables before the study, and learning curve needed. - Assess usability and appropriateness of the devices. - Identify pain points of dealing with the devices. | 1. Had you ever used a wearable device (activity tracker, health tracker, etc.) prior to this study?   *Prompts: What type of device was it? How long did you use it? If you stopped using it, why did you stop?*   1. How do you find the appearance and comfort of the device?   *Prompts: If you had to rate it between 1-10, how would you rate the device used in the study? Why that rating? What features did you like about it, and what features did you dislike?*   1. Do you ever have any difficulties using the device? Can you give me some examples?   *Prompts: During charging? During syncing? During setup? Were you able to wear the device for long periods of time or whilst you were sleeping without it causing any annoyance or discomfort? Did level of comfort impact on your choice to wear the device?*   1. Have you ever chosen not to wear the device? Why was that, and why/when did you start wearing it again? 2. Are you ever concerned about what people thought of you wearing the device? Can you give me an example? |
| **Experience with at home CRP devices.**   - Assess familiarity at home CRP devices before the study, and learning curve needed. - Assess usability and appropriateness of the devices. - Identify pain points of dealing with the devices. | 1. Had you ever used at home CRP test kit, or similar at home medical device prior to this study?   *Prompts: What type of device was it? How long did you use it? If you stopped using it, why did you stop?*   1. How do you find the difficulty of using the device? Can you give me some examples of any difficulties you encountered?   *Prompts: If you had to rate it between 1-10 on the ease of using the test kit, how would you rate it? Why that rating? What features did you like about it, and what features did you dislike?*  *Are there any difficulties during the withdrawal of blood? Are the instructions booklet and the help given to learn how to use the device sufficient? Are there any discomfort when you are using the device?*   1. Have you ever chosen not to use the at home CRP anymore? Why was that? |
| **Assessment of health anxiety issues related to technology use within the study and probes to understand associated ‘help’ seeking behaviors.** | 1. Has there been anything about the mobile device that has made you uncomfortable (concerned, worried)? Please describe.   *Prompt: If so, what did you do about being made uncomfortable? Did you involve anyone else e.g GP, specialist, family member. Please would you tell me about this. Have you any thoughts on ways that would have helped to make you less uncomfortable?*   1. Did the mobile device/app make you less concerned about anything? Please tell me about this.   *Prompt: If you had not got the device what would you have done? Might you have involved others e.g. GP, specialist, nurse, family member. How has this impacted you more generally?*   1. Has there been anything about the wearable/at home devices that has made you uncomfortable (concerned, worried)? Please describe.   *Prompt: If so, what did you do about being made uncomfortable? Did you involve anyone else e.g GP, specialist, family member. Please would you tell me about this. Have you any thoughts on ways that would have helped to make you less uncomfortable?*   1. Did the wearable make you less concerned about anything? Please tell me about this.   *Prompt: If you had not got the device what would you have done? Might you have involved others e.g. GP, specialist, nurse, family member. How has this impacted you more generally?* |
| **Data Privacy & Sharing**   - Assess perceived value of data - Identify concerns about being monitored throughout the study | 1. Was there anything about being measured during the study that made you uncomfortable? What was it and why? 2. Would it have been useful for you to receive information about the data collected throughout the study? Why or why not?   *Prompts: Would it make you more or less likely to participate in a future study? Do you have experience of using any other apps or symptom feedback systems? If you would look at feedback, how would you like to receive this? Should it be optional? Provided in the app? Should it be visual, with graphs, or verbal feedback? What kinds of graphs are useful? How often should it be provided?*   1. Are there any problems about how your data is being used that you would like to raise with us?   *Prompt: Do you not want us to include it in any analysis or papers, even though they are anonymised? Are there any extra steps that you would like us to take?* |
| **Closing & Improvements needed in Study Design** | 1. Do you find that the benefits of taking part outweigh the costs of taking part? Or the other way round that the costs outweigh the benefits? 2. Do you think there is value in this approach to gathering data about your health? How would you describe that value? 3. As you continue to participate for a longer period, what would need to be different? 4. Is there anything else you would like to say? |
