# Supplementary material for: Feasibility of remote measurement in intensive longitudinal data collection for rheumatoid arthritis patients commencing a new treatment
Source: Rheumatol Adv Pract. 2025 Jul 7;9(3):rkaf078. doi: 10.1093/rap/rkaf078 (PMC12375405; doi:10.1093/rap/rkaf078)
Supplement: rkaf078_Supplementary_Data [file rkaf078_supplementary_data.zip › Supplementary Data.docx]

Supplementary Data S1: EMA Survey Questions

Q1 Please rate how much you felt each of the following in the last hour. Responses are all 0-10 scale, with 0 = none and 10 = extreme

|  | None | Moderate | Extreme |
| --- | --- | --- | --- |

|  | 0 | 1 | 2 | 3 | 4 | 5 | 6 | 7 | 8 | 9 | 10 |
| --- | --- | --- | --- | --- | --- | --- | --- | --- | --- | --- | --- |

| Pain () | 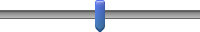 |
| --- | --- |
| Joint stiffness () | 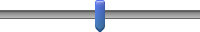 |
| Fatigue () | 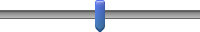 |
| Sad () | 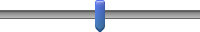 |
| Lonely () | 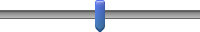 |
| Anxious () | 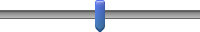 |
| Irritable () | 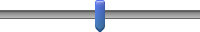 |
| Relaxed () | 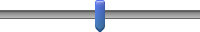 |
| Content () | 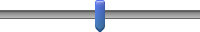 |
| Enthusiastic () | 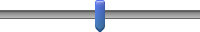 |
| Cheerful () | 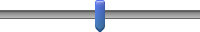 |

Supplementary Data S2: Daily Survey Questions

Q1 Please rate how much you felt each of the following in the last hour. Responses are all 0-10 scale, with 0 = none and 10 = extreme

|  | None | Moderate | Extreme |
| --- | --- | --- | --- |

|  | 0 | 1 | 2 | 3 | 4 | 5 | 6 | 7 | 8 | 9 | 10 |
| --- | --- | --- | --- | --- | --- | --- | --- | --- | --- | --- | --- |

| Pain () | 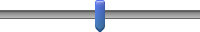 |
| --- | --- |
| Joint stiffness () | 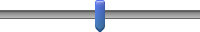 |
| Fatigue () | 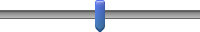 |
| Sad () | 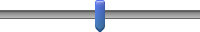 |
| Lonely () | 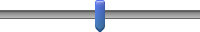 |
| Anxious () | 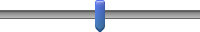 |
| Irritable () | 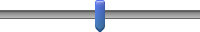 |
| Relaxed () | 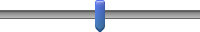 |
| Content () | 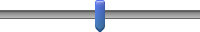 |
| Enthusiastic () | 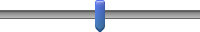 |
| Cheeful () | 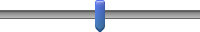 |

Q2 The following scale will be regarding your well-being today. The response is from a score of 0 to 10, with 0 being none and 10 being extreme.

|  | None | Moderate | Extreme |
| --- | --- | --- | --- |

|  | 0 | 1 | 2 | 3 | 4 | 5 | 6 | 7 | 8 | 9 | 10 |
| --- | --- | --- | --- | --- | --- | --- | --- | --- | --- | --- | --- |

| How much have your joint or muscle symptoms bothered you today? () | 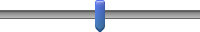 |
| --- | --- |
| Did you experience any problems physically performing the tasks you were doing today? () | 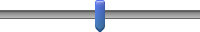 |
| How much enjoyment did you experience from your activities today? () | 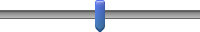 |
| How satisfied were you with the amount of social interactions you had today? () | 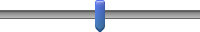 |
| How supported by others did you feel today? () | 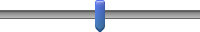 |

Q3 How many hours did you sleep last night?

- 4 hours or less (1)
- 4.5 hours (2)
- 5 hours (3)
- 5.5 hours (4)
- 6 hours (5)
- 6.5 hours (6)
- 7 hours (7)
- 7.5 hours (8)
- 8 hours (9)
- 8.5 hours (10)
- 9 hours (11)
- 9.5 hours (12)
- 10 hours or more (13)

Q4 What was the quality of your sleep last night?

|  | Very poor | Excellent |
| --- | --- | --- |

|  | 0 | 1 | 2 | 3 | 4 | 5 | 6 | 7 | 8 | 9 | 10 |
| --- | --- | --- | --- | --- | --- | --- | --- | --- | --- | --- | --- |

| Sleep quality () | 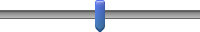 |
| --- | --- |

Q5 Did you nap during the day?

- Yes (1)
- No (2)

Q6 What was your highest level of physical activity today?

- Resting (e.g. napping) (1)
- Sitting (e.g. watching tv, working) (2)
- Standing (e.g. cooking) (3)
- Walking slowly (e.g. doing housework, gardening, yoga) (4)
- Walking briskly (e.g. fast walk as a form of exercise outside, cycling) (5)
- Moderate intensity exercise (e.g. carrying light loads, jogging, social tennis) (6)
- Vigorous or high intensity exercise (e.g. swimming, HIIT (high intensity interval training) workouts, sprints) (7)

Q7 For how long did you wear your fitbit today?

- Less than 1 hour or not at all (1)
- 1 hour to 3 hours (2)
- 3 hours to 6 hours (3)
- 6 hours to 9 hours (4)
- More than 9 hours (5)

Q8 Did you undertake any strenuous physical activity today while not wearing your Fitbit?

- Yes (1)
- No (2)

Supplementary Data S3: Qualitative Codebook

| **Codes** |
| --- |
|  |
| **Affective Attitude** |
| Comfortable with data collected |
| Surveys valuable/helpful to patient |
| Fitbit valuable/helpful to patient |
| Concerns with data collected |
| Helping others |
| Positive study experience |
| **Perceived effectiveness** |
| **Data capture** |
| Missing surveys |
| Flexibility of surveys |
| No problems using surveys |
| Familiarity with trackers |
| Close timing of surveys |
| Questions wording/format |
| Accuracy of Fitbit |
| Frequency of Fitbit use |
| External contributing factors- to survey data |
| External contributing factors- to Fitbit data |
| Missing capture of symptoms |
| Technology barriers |
| **Real world use** |
| No changes in behaviour |
| Sleep awareness |
| Sleep behaviour change |
| Physical activity awareness |
| Physical activity behaviour change |
| General health awareness - positive |
| Mental health awareness |
| Closeness to medical team |
| Useful to care team |
| Useful when changing medication |
| Reassurance from close monitoring |
| **Perceived burden and costs** |
| High survey burden |
| Low survey burden |
| No increased symptom concern- survey |
| Increased symptom concern - survey |
| Adapting to surveys |
| Increased health concern - fitbit |
| No increased health concern - fitbit |
| Low tracker burden |
| Adapting to tracker |
| Comfort and wearability of tracker |
|  |
|  |
| Definition of themes and subthemes from thematic analysis |
|  |
| \| **Theme** \| **Sub-Theme** \| **Definition** \| \| --- \| --- \| --- \| \| **Affective Attitude** \| - \| Participants reported feelings towards the research experience overall. \| \| **Perceived Burden & Costs** \| - \| Perceived participant effort and negative impacts associated with completing the study, including participants ability to understand the data collection methods. \| \| **Perceived Effectiveness** \| Data capture \| The extent to which data collection was perceived to achieve its purpose of capturing useful patient information. \| \|  \| Real world use \| Participants feedback on implementing EMA data collection into real-world healthcare. \| |
|  |
|  |
|  |

Figure S1. Self reported and FitBit data for activity


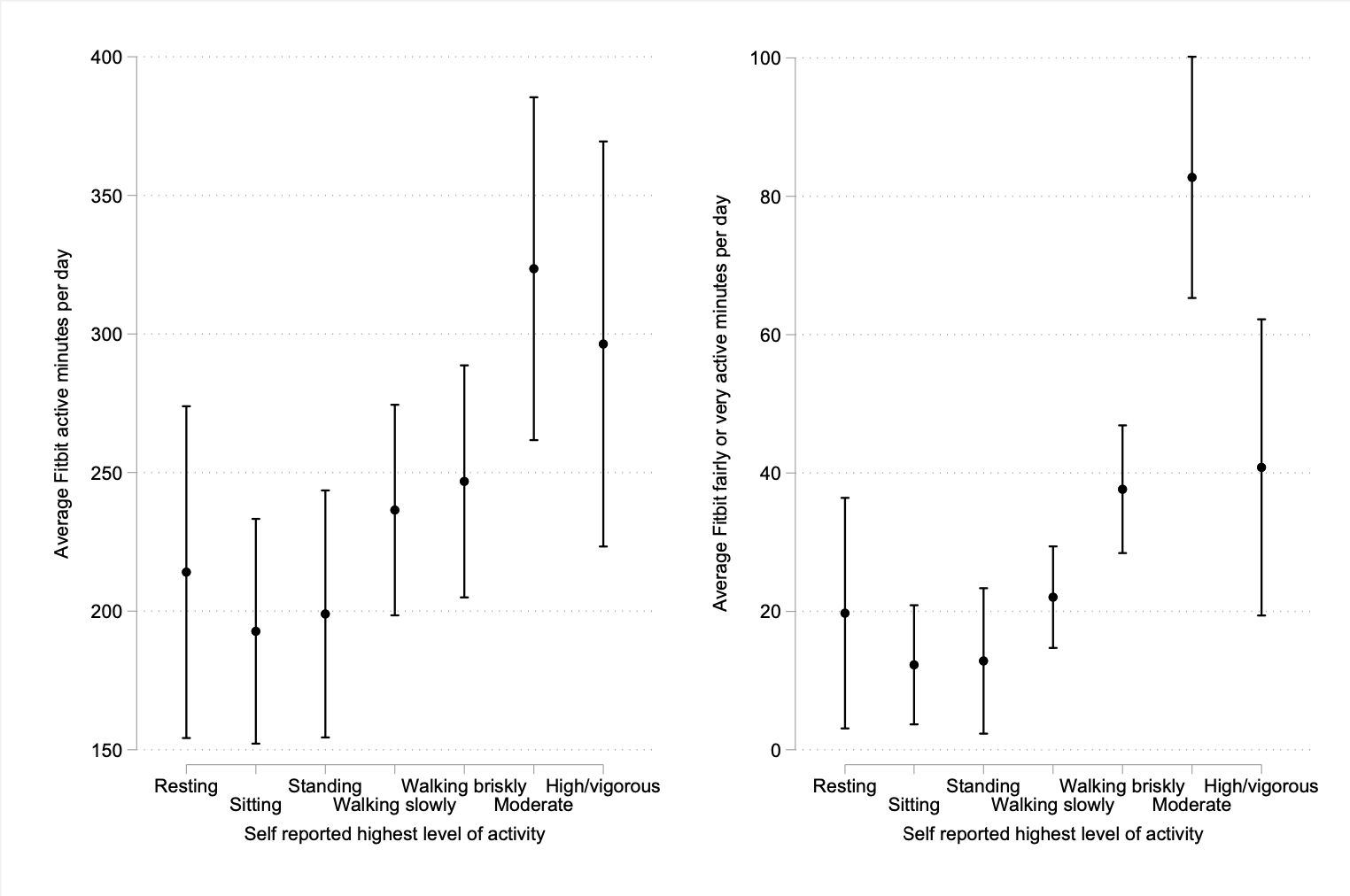


Figure S2. Self reported and FitBit data for Sleep


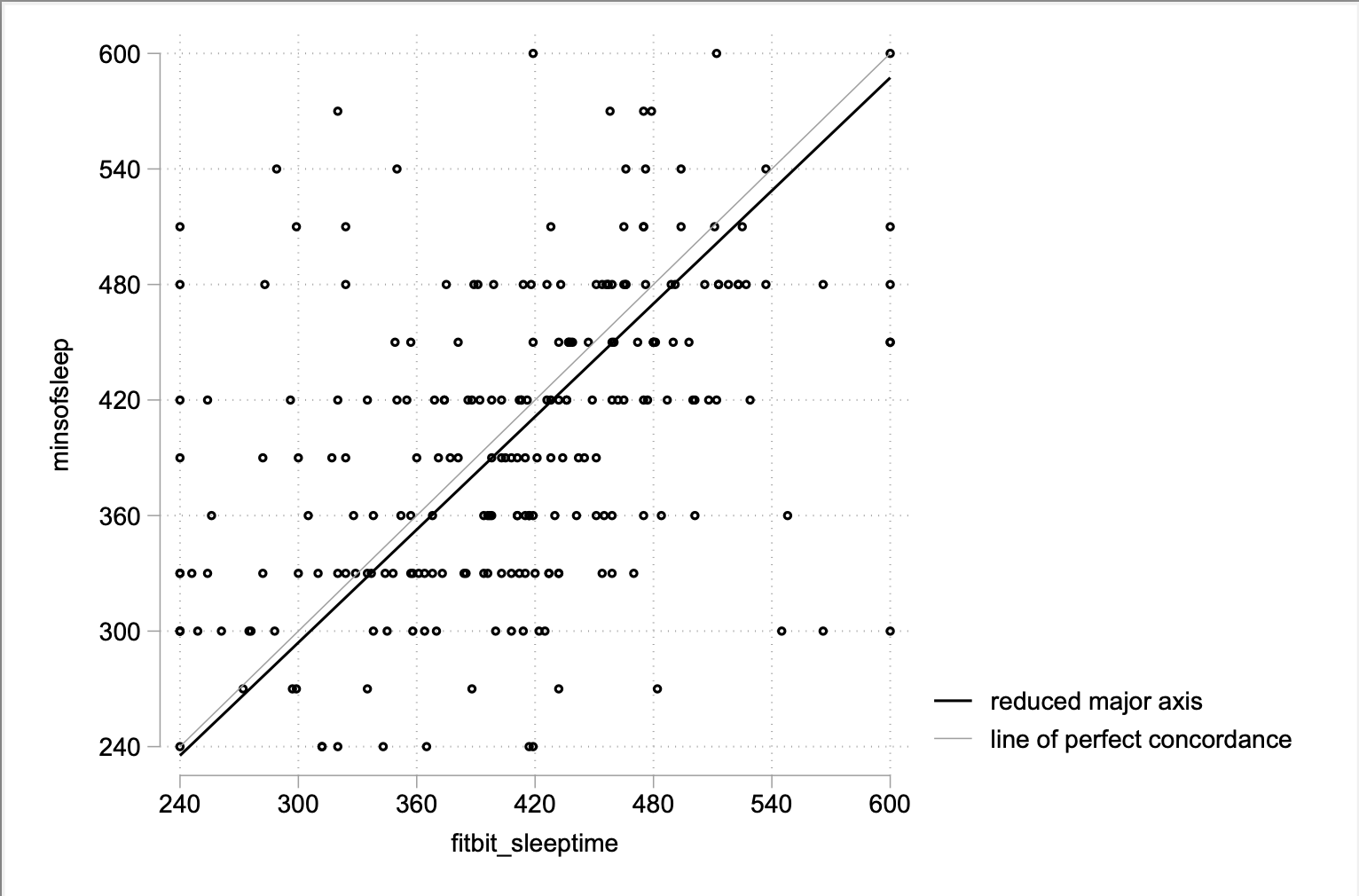


| Variable | Mean difference post versus pre | SE | Z | p-value | LCL | UCL |
| --- | --- | --- | --- | --- | --- | --- |
| Mean pain | -0.97 | 0.12 | -8.22 | 0.000 | -1.20 | -0.74 |
| Mean joint stiffness | -0.85 | 0.12 | -7.32 | 0.000 | -1.07 | -0.62 |
| Mean fatigue | -0.44 | 0.13 | -3.44 | 0.001 | -0.70 | -0.19 |
| Variability (SD) pain | -0.22 | 0.06 | -3.88 | 0.000 | -0.33 | -0.11 |
| Variability (SD) pain | -0.26 | 0.06 | -4.52 | 0.000 | -0.37 | -0.15 |
| Variability (SD) fatigue | -0.31 | 0.07 | -4.61 | 0.000 | -0.45 | -0.18 |


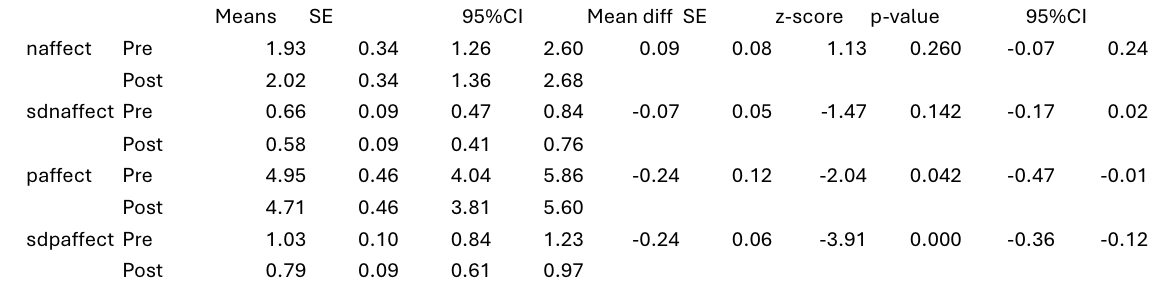


Table S2: Mean difference and variability of affect pre and post treatment

Table S1: Mean difference and variability of somatic symptoms pre and post treatment
